# Continuous theta burst stimulation for drug-resistant epilepsy

**DOI:** 10.3389/fnins.2022.885905

**Published:** 2022-08-17

**Authors:** Sofie Carrette, Paul Boon, Debby Klooster, Annelies Van Dycke, Evelien Carrette, Marijke Miatton, Robrecht Raedt, Jean Delbeke, Alfred Meurs, Kristl Vonck

**Affiliations:** ^1^Department of Neurology, Institute for Neuroscience, Ghent University Hospital, Ghent, Belgium; ^2^Department of Electrical Engineering, Eindhoven University of Technology, Eindhoven, Netherlands; ^3^Department of Neurology, Sint-Jan General Hospital, Bruges, Belgium

**Keywords:** neurostimulation, theta burst stimulation, transcranial magnetic stimulation (repetitive), epilepsy, safety, treatment

## Abstract

**Introduction:**

Repetitive transcranial magnetic stimulation (rTMS) may have anti-epileptic effects, especially in patients with neocortical lesions. Initial clinical trials demonstrated that the duration of the seizure reducing effect is relatively short-lived. In the context of a chronic condition like epilepsy, theta burst stimulation (TBS) may represent a potential solution in optimizing treatment practicality and durability as it was demonstrated to be associated with longer-lasting after-effects. TBS has been studied extensively in diverse neuropsychiatric conditions, but a therapeutic TBS protocol has not previously been applied in epilepsy patients.

**Materials and methods:**

We performed a prospective open-label pilot study of 4-day accelerated continuous TBS (cTBS) treatment in patients with neocortical drug-resistant epilepsy (DRE). A treatment session consisted of 5 cTBS trains, each comprising 600 pulses presented in 50 Hz triplet bursts every 200 ms, delivered at 10-min intertrain-intervals, targeted over the epileptic focus (EF) using a neuronavigation-guided figure-of-8 coil. Safety and feasibility, and seizure frequency were assessed as primary and secondary endpoints, respectively, over a 4-week baseline period, a 1-week treatment period and a 7-week follow-up period, using adverse event logging, electro-encephalography, cognitive, and psychological questionnaires and a seizure diary kept by the patients and/or caregivers.

**Results:**

Seven subjects (4M:3F; median age 48, interquartile ranges 25) underwent the treatment protocol. Adverse events were reported in all subjects but were mild and transient. No clinical or electrographic seizures were evoked during or immediately following stimulation. No deterioration was found in cognition nor in psycho-emotional well-being following treatment. Treatment burden was acceptable, but seems to depend on clinical effect, duration of ongoing effect and stimulation site. Median weekly seizure frequency and ratio of seizure-free weeks did not change significantly in this small patient cohort.

**Conclusion:**

We report the results of the first ever trial of cTBS as a treatment for neocortical DRE. A 4-day accelerated cTBS protocol over the EF appears safe and feasible. Although the design and sample size of this open-label pilot study is unfit to reliably identify a therapeutic effect, results encourage further exploration of cTBS as an anti-epileptic treatment and potential optimization compared to conventional rTMS in a dedicated randomized controlled trial. (clinicaltrials.gov: NCT02635633).

## Introduction

Novel and non-invasive neurostimulation modalities are under investigation as a third line treatment option for patients with drug-resistant epilepsy (DRE). Transcranial magnetic stimulation (TMS) is a non-invasive neurostimulation modality first described by Barker et al. (1985). The technique is based on Faraday’s law of electromagnetic induction where a magnetic field acts as a “carrier” to transmit an electrical current from the stimulation coil to the brain (Barker, 2002; Klooster et al., 2016). When administered in particular repetitive paradigms, repetitive TMS (rTMS) may increase or decrease cortical excitability (Rossini and Rossi, 2007). The direction of the induced effects is related to the frequency or pattern of the applied stimuli, resulting in the development of various clinically applied protocols such as low- or high-frequency rTMS, theta burst stimulation (TBS), paired pulse, or quadripulse stimulation (Rossini and Rossi, 2007; Hamada and Ugawa, 2010).

In the context of epilepsy, the pathognomonic cortical hyperexcitability is targeted by an inhibitory rTMS paradigm. Three meta-analyses reported a significant seizure reducing effect, primarily in patients with cortical dysplasia and neocortical epilepsy when low-frequency rTMS was delivered over the epileptic focus (EF; Hsu et al., 2011; Cooper et al., 2018; Mishra et al., 2020). The duration of the seizure reducing effect was relatively short-lived (Mishra et al., 2020), which is disadvantageous in the context of a chronic condition.

Theta burst stimulation may represent a potential solution in optimizing treatment practicality and effect duration. This novel stimulation paradigm seems able to induce longer-lasting after-effects with less pulses at a lower stimulation intensity (Huang et al., 2005). In practice, an inhibitory continuous TBS (cTBS) train, consisting of 600 pulses presented in 50 Hz triplet bursts every 200 ms, is completed in 40 s compared to the 30–60 min stimulation in conventional 1 Hz rTMS. As a result, TBS has been studied extensively in diverse neuropsychiatric conditions, with FDA approval of intermittent TBS (iTBS) over the left dorsolateral prefrontal cortex to treat major depressive disorder (O’Reardon et al., 2007; Lisanby et al., 2009; Levkovitz et al., 2015). Further optimization strategies have been described more recently, using accelerated (Desmyter et al., 2016) and spaced (Williams et al., 2018) TBS protocols, applying multiple stimulation trains per day as a compact high-intensity treatment protocol. These protocols have not been associated with increased adverse events nor with an increased risk of seizure induction (Rossi et al., 2021). Experience in epilepsy patients is scarce and TBS as an anti-epileptic treatment has not yet been investigated in clinical trials.

In this pilot study, we investigated the safety and feasibility of neuronavigation-guided cTBS in patients with neocortical DRE. As a secondary outcome parameter, we investigated the seizure reducing effect of cTBS during long-term follow-up.

## Materials and methods

### Participants

Patients were recruited at the Reference Center for Refractory Epilepsy (RCRE) at Ghent University Hospital in Belgium. Eligible participants, aged between 18 and 65 years, had DRE with a well-defined neocortical ictal onset zone based on a standardized presurgical evaluation and a baseline seizure frequency ≥ 4 seizures/month in the 6 months prior to inclusion. Additional requirements were a stable drug regimen for at least 2 months with therapeutic compliance in the past, IQ > 70 and ability to complete a seizure diary by the patient or his/her caretaker. Exclusion criteria were a history of psychogenic non-epileptic seizures, pregnancy, short-term birth wish or childbearing age without adequate birth control and the presence of intracranial metal hardware, pacemaker, implantable cardioverter-defibrillator, permanent medication pumps, cochlear implants, or deep brain stimulation. Vagus nerve stimulation (VNS) was not considered a contra-indication provided that adequate distance between the coil and the implanted material could be maintained.

### Study design

**FIGURE 1:**
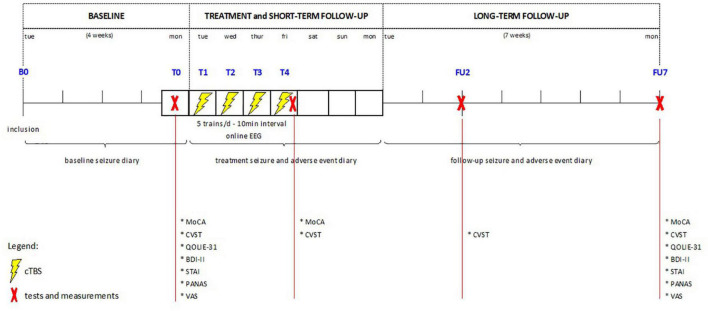
Overview of study design. cTBS, continuous theta burst stimulation; EEG, electroencephalography; MoCA, Montreal Cognitive assessment; CVST, comuterized visual searching task; QOLIE-31, quality of life in epilepsy-31; BDI-II, Beck depression inventory version II; STAI, state-trait anxiety inventory; PANAS, positive affect negative affect scale; VAS, visual analogue scale.

The trial (clinicaltrials.gov identifier: NCT02635633) is a prospective open-label pilot study of cTBS, approved by the local Ethics Committee of Ghent University Hospital. Informed consent was obtained from patients and/or their caregivers prior to inclusion. Figure 1 shows a visual representation of the study protocol. Intervention consisted of 4 consecutive days of spaced cTBS delivered over the neocortical EF as determined by prior presurgical evaluation.

Tolerability, safety and seizure frequency were assessed over a 4-week baseline period (B), a 1-week treatment period (T), and a 7-week follow-up period (FU), using adverse event logging, dedicated cognitive and psycho-emotional questionnaires and a seizure diary kept by the patients and/or their caregivers.

### Repetitive transcranial magnetic stimulation procedure

Continuous theta burst stimulation was performed using a MagPro X100 stimulator with a static cooled 65 mm figure-of-8 stimulation coil (*Magventure, Farum, Denmark*). Precise targeting of the EF was achieved using an online neuronavigation system for frameless stereotaxy (*Localite, Bonn, Germany*) with a Polaris infrared camera (*Northern Digital Inc., Waterloo, Canada)* based on structural MRI imaging of the individual subject.

A treatment session consisted of 5 cTBS trains, each comprising 600 pulses presented in 50 Hz triplet bursts every 200 ms during 40 s, delivered at 10-min intertrain-intervals. Per session 3000 stimuli were delivered with a total of 12.000 stimuli over the entire 4-day treatment. Stimulation intensity was set relative to the resting motor threshold (rMT), determined at baseline and used as the reference throughout the week. rMT was determined at the abductor pollicis brevis (APB) motor hotspot ipsilateral to the EF, using the “threshold hunting method.” This is an adaptive method based on Parameter Estimation by Sequential Testing and Maximum Likelihood regression (Awiszus, 2003; Silbert et al., 2013). A correction for different coil-cortex distance at the target area (EF) compared to the motor hotspot (HS) was applied, according to the following formula (Stokes et al., 2013), in which *D*_*EF*_ represents the coil-cortex distance at the EF, and *D*_*HS*_ the distance at the APB hotspot.


$$\text{AdjMT}=\text{rMT}+k\times \left(D_{\text{EF}}-\,D_{\text{HS}}\right)$$

[AdjMT and rMT expressed in % of maximal stimulator output (MSO); D_*EF*_ and D_*HS*_ in mm; *k* = 2.7%/mm].

As a result, stimulation intensity was individually determined and set at 80% of AdjMT. In cases where the rMT exceeded the capacity of the stimulator output, stimulation intensity was set at 100% MSO.

Throughout the stimulation session patients were seated in a reclined chair and provided with hearing protection. Online neuronavigation allows continuous coil tracking relative to the target area and adjustment of the coil position during the stimulation train if needed.

### Electro-encephalography

Prior to the first cTBS session, 21 TMS-compatible AgCl-coated plastic EEG electrodes (*MedimaxTech, Compton, United States*) were placed on the scalp according to the International 10–20 System. Reference and ground electrode were placed on the forehead. Electrode impedances were kept below 20 kΩ. Electrodes remained in place throughout all 4 stimulation sessions, allowing online EEG acquisition using DC amplifiers (*BrainAmp MRplus, Brainproducts GmbH, Gilching, Germany*). The obtained EEG signal was amplified, filtered (DC-1,000 Hz), digitized with 0.5 μV resolution and a sampling rate of 5,000 Hz (*Brainvision Recorder, Brainproducts GmbH, Gilching, Germany*) and stored for offline processing.

### Assessments

#### Safety

##### Adverse events

Adverse event monitoring was performed as follows: (1) during and immediately following stimulation, subjects were visually inspected and asked for discomfort or other complaints; (2) subjects were asked to log any complaint developing later on in a diary. These were discussed at each study contact and categorized for potential relation to cTBS as *likely*, *unlikely* or *uncertain*, based on timing and stimulation target.

##### Transcranial magnetic stimulation-related seizures

Provocation or exacerbation of seizures by cTBS was assessed as follows: (1) subjects were closely monitored by the investigator (SC) during delivery of a stimulation train. In case of seizure induction, stimulation would be stopped, with close assessment of the subject, comparison to habitual seizure semiology and logging of the event; (2) online EEG acquisition during each stimulation session with retrospective evaluation for (sub-)clinical ictal discharges (KV); and (3) subjects and/or their caretakers were asked to log all seizures occurring during the treatment week in a dedicated diary. Each reported seizure was discussed the following day or at follow-up and evaluated for relation to the stimulation train based on semiology and stimulation target. Any seizure occurring during treatment week was categorized as *likely*, *unlikely* or *uncertanly* related to cTBS.

##### Cognition

Cognition was assessed using the Montreal Cognitive Assessment (MoCA) and the Computerized Visual Searching Task (CVST). The MoCA is a validated screening tool to assess several cognitive domains in a quick and easy way, including *short-term memory recall, visuospatial abilities*, multiple aspects of *executive functions*, *attention, concentration, and working memory*, *language* and *orientation in time and space*. Each of the three validated versions for short-term consecutive testing (original version; parallel version 7.2; parallel version 7.3) was performed prior to treatment (T0), following final stimulation session (T4) and at the end of the 7-week follow-up period (FU7), respectively. The CVST is a computerized test that consists in finding among a set of 24 the grid pattern that matches the one presented at the center of the screen. A different pattern is presented twenty-four times and the participant is asked to react as fast as possible. CVST assesses the speed of *central information processing*. Accuracy and speed of response are recorded per grid and the average response time is used for analysis. CVST is performed prior to treatment (T0), following final stimulation session (T4), at 2 weeks (FU2) and at the end of the follow-up period (FU7).

##### Psycho-emotional well-being

Psycho-emotional well-being was evaluated using different questionnaires at T0 and FU7. *Quality of life* (QoL) is assessed using the Quality of Life in Epilepsy-31 (QOLIE-31), providing a calculated QoL score based on several subdomains as well as a subjective perception of QoL by the subject using a visual analogue scale (VAS) ranging from 0 to 100, with 100 being the best possible QoL. *Depression* is rated using the beck depression inventory (BDI-II). Scoring of *anxiety* uses the state-trait anxiety inventory (STAI), with S-STAI representing the state of anxiety at the time of questioning and T-STAI the trait of the respondent in general. *Positive or negative affect* is assessed using the positive affect negative affect scale (PANAS) and general *subjective wellbeing* using a VAS (0–100), with 100 being the best possible well-being.

#### Feasibility/tolerability

Tolerability is a prerequisite for cTBS as a treatment in clinical practice. It is evaluated using a VAS of treatment burden, drop-out rate and willingness to repeat treatment in an extended stimulation protocol.

#### Anti-epileptic effect

The anti-epileptic effect of cTBS is evaluated by comparing the reported seizure frequency during baseline and follow-up. The participant and/or a caretaker was asked to log every seizure in a dedicated diary, including information on semiology, severity and comparison to habitual seizures when relevant. At each follow-up contact (FU2 and FU7), the diary was evaluated for completeness or dubious reporting and discussed when necessary. In case of multiple seizure types in a single subject, the sum of all seizure types was considered for statistical analysis. Incomplete reporting of a seizure type at any time point resulted in omission of this type from overall analysis. The following outcome parameters were derived from the raw seizure diary data: (1) absolute number of reported seizures per week; (2) median number of seizures per week during baseline (4 week period) and follow-up (7 week period); (3) ratio of seizure-free weeks during baseline (number of seizure-free weeks divided by 4) and follow-up (number of seizure-free weeks divided by 7); and (4) response rate (percentage of median seizure frequency reduction or increase during follow-up compared to baseline).

### Statistical analysis

Given the small sample size of this pilot study, data are primarily reported in a descriptive way. Data evaluation was performed on the single subject as well as group level. In this last case median values and interquartile ranges (IQR) are used in order to minimize distorting outlier effects. Statistical analysis was performed using SPSS^®^ (*Version 28, IBM^®^ SPSS^®^ Statistics, United States*), performing two-sided non-parametric tests for two related samples (*Wilcoxon matched-pairs signed-ranks test*) or more related samples (*Friedman test*), with power β = 0.80 and α < 0.05 as the margin of significance.

## Results

### Patient characteristics

Seven subjects (4M:3F; median age 48, IQR 25) were included in the study between August 2015 and November 2017. Demographics and clinical data of the patients are given in Table 1. All subjects had refractory unifocal epilepsy with a median epilepsy duration of 15 (IQR 16.5) years. They had failed on average 7 (IQR 2.5) anti-epileptic drugs (AEDs) and were actively treated with 4 (IQR 2) AEDs. One participant was additionally treated with VNS. The EF was located in the left frontal lobe (*n* = 5), right frontal lobe (*n* = 1), and in the lateral temporal lobe (*n* = 1). Etiologies were: low-grade tumor (*n* = 2), focal cortical dysplasia (*n* = 1), post-hemorrhage structural lesion (*n* = 1), and cryptogenic (*n* = 2).

**TABLE 1:** Demographic and clinical data of the study population.

Participant	Age	Gender	Duration of epilepsy (years)	Previously failed AEDs (number)	Current AEDs	Presurgical evaluation	Epileptogenic focus	Etiology	Ictal semiology	Vagus nerve stimulation
1	51	M	14	6	CBZ, LEV, VPA	VEM, MRI, fMRI, DTI, FDG-PET, NPO	right frontal (motor cortex, paramedian)	oligodendroglioma grade 2 in right paramedian motor cortex	(reflex) myoclonia of the left leg	N
									focal motor seizure of the left leg or left hemisoma	
									focal-to-bilateral tonic-clonic seizure	
2	26	M	17	6	LEV, CBZ, PGB, LZP	EEG, MRI	left frontal (motor cortex, handknob)	multicystic fibrillary astrocytoma in left primary motor cortex	focal motor seizure of the right trunk and arm	N
									focal motor seizure of the right hemisoma	
									focal motor seizure with impaired consciousness and postictal paresis	
3	48	F	38	16	CBZ, CLB, TPM, LCS, PHT	VEM, MRI, fMRI, ictal SPECT	left frontal (motor cortex, paramedian)	cryptogenic	reflex myoclonia of the right leg	N
									focal motor seizures of the right leg, onset during sleep	
4	55	F	8	7	LEV, LCS	VEM, MRI, fMRI, FDG-PET, NPO, Wada	left temporal (auditory cortex)	lesional post-hemorrhagic left temporal	focal auditory seizures	N
									focal auditory seizures and speech arrest	
									focal auditory seizures, speech arrest and impaired consciousness	
									prolongued episodes of impaired speech	
5	53	M	42	10	CLB, VGB, LEV, PHT, PB	VEM, MRI, fMRI, FDG-PET, ictal SPECT, NPO, hdEEG-ESI, MEG, MRI post-processing	left frontal (premotor cortex)	focal cortical dysplasia left premotor cortex	focal motor seizure of right arm, often clustered	N
									focal-to-bilateral tonic-clonic seizure	
6	20	M	15	7	LEV, LTG, TPM	*performed in Netherlands*	left frontal (motor cortex, handknob)	focal cortical dysplasia left frontal	nocturnal focal motor seizure of right arm and leg	Y
7	28	F	5	8	LEV, DZP, TPM, LTG, PER	VEM, MRI, NPO, EEG-ESI, MEG	left frontal (inferior frontal gyrus)	cryptogenic post-encephalitis	focal sensory seizures of right hemicorpus	N
									focal sensorimotor seizures of right hemicorpus with speech impairment	

AEDs, anti-epileptic drugs; M, male; F, female; CBZ, carbamazepine; LEV, levetiracetam; VPA, valproic acid; PGB, pregabalin; LZP, lorazepam; CLB, clobazam; TPM, topiamate; LCS, lacosamide; PHT, phenytoine; VGB, vigabatrin; PB, phenobarbital; LTG, lamotrigine; DZP, diazepam; PER, perampanel; VEM, video-EEG monitoring; MRI, magnetic resonance imaging; fMRI, functional magnetic resonance imaging; DTI, diffusion tensor imaging; FDG-PET, fluorodesoxyglucose positron emission tomography; NPE, neuropsychological evaluation; EEG, electro-encephalography; SPECT, single photon-emission computed tomography; ESI, electrical source imaging; and MEG, magneto- encephalography.

### Safety

#### Adverse events

Table 2 summarizes the adverse events reported throughout the study. These are subdivided based on temporal relation to the delivered stimulation trains and rated for causal relation to cTBS.

**TABLE 2:** Adverse events reported during treatment week and follow-up.

Adverse event	Stimulation target	Number of subjects (%)	Relation to cTBS		
			Likely	Unlikely	Uncertain
**Reported during stimulation**					
Local sensation underneath stimulation coil	All	7	√		
Sensation in right hemisoma, starting from the mouth (resembling habitual prodromal feeling)	Left paramedian motor cortex	1	√		
Jaw contractions during stimulation	Left temporal cortex; left frontal inferior gyrus	2	√		
Dizziness (bouts of seconds)	Left paramedian motor cortex	1	√		
**Reported in between stimulation trains**					
Reflex myoclonic seizure following sudden sound	Right paramedian motor cortex	1		√	
**Reported immediately following stimulation**					
Headache, pain medication required		1	√		
Headache, no pain medication required		2	√		
Lateralized feeling right hemisoma (habitual prodromal)	Left paramedian motor cortex	1	√		
**Reported during treatment week, not in immediate temporal relation to stimulation**					
Headache, pain medication required		2	√		
Headache, no pain medication required		1	√		
Heavy feeling right arm (habitual prodromal feeling)	Left handknob motor cortex	1			√
Fatigue	Left paramedian motor cortex; left temporal cortex	2			√
Hot flushes	Left temporal cortex	1			√
Near fall, unclear conditions	Left temporal cortex	1			√
Diplopia	Left handknob motor cortex	1		√	
Reflex myoclonic seizure	Right paramedian motor cortex; left paramedian motor cortex	2		√	
**Reported during follow-up**					
Hyperacusis (onset during treatment week)	Left frontal inferior gyrus	1	√		
Fatigue	Left temporal cortex	1			√
Tremor and dizziness (episode)	Left temporal cortex	1			√
Headache, pain medication required, also described during baseline	Left paramedian motor cortex; left temporal cortex	2			√
Dizziness (episode), also described during baseline	Left temporal cortex	1		√	
Joint pain right arm	Right paramedian motor cortex	1		√	
Joint pain right foot	Left paramedian motor cortex	1			√

##### Adverse events during, immediately following and shortly after stimulation

All subjects (7/7) reported a local sensation underneath the stimulation coil during the cTBS train. In two subjects (subject 4 and subject 6) the stimulation train was associated with jaw contractions ipsilateral to the stimulation site. Stimulation site was left lateral temporal and left inferior frontal cortex in these subjects. One subject (subject 3) reported a particular sensation in the right hemisoma, starting from the mouth region, only during stimulation trains on the second and the third treatment day. On treatment day 2, this sensation was also reported to be ongoing after the treatment session and was described as a habitual prodromal feeling of a focal motor seizure, which did not occur. The target area in this subject was the left paramedian primary motor cortex. This same subject reported short bouts of dizziness during stimulation. In view of the close temporal relationship with cTBS delivery, all these adverse events were considered to be related to cTBS.

Three subjects reported headache immediately following stimulation (not in all sessions), most often mild in severity, short-lived and not requiring pain medication in 2/3. Three subjects also reported onset of headache at a later time point following stimulation, requiring pain relief in two. This was also considered related to cTBS.

One subject (subject 2) reported a heavy feeling in the right arm upon awakening on the fourth treatment day, typically prodromal to a seizure, but no seizure occurred. Relation to cTBS was considered uncertain. Two subjects (subject 3 and 4) reported fatigue during the treatment week. Subject 4 also reported a few instances of hot flushes and a near fall while walking, in unclear conditions but without loss of consciousness or other neurological symptoms. Relation to cTBS for these AEs was also considered uncertain.

Subject 2 reported an episode of diplopia on the way home from the second stimulation session. This subsided after having a meal and is therefore suggestive of a hypoglycemic event and considered unlikely related to cTBS.

##### Adverse events during follow-up

One subject (subject 7) reported hyperacusis, which developed over the course of the treatment week (but was not reported at that time) and lasted until a few days thereafter. Stimulation target in this subject was the left inferior frontal gyrus. The relation to cTBS was considered likely.

Over the course of the 7-week follow-up period, two subjects (subject 3 and subject 4) reported headache. In subject 4 this headache was unchanged compared to a high baseline frequency. In subject 3, headache developed the day after the final treatment session and persisted for 1 week. Headache phenotype was sporadically known to the patient, but onset may be related to the administration of cTBS.

Two subjects reported joint pain, the right arm in subject 1 and the right foot in subject 3. Relation to cTBS in subject 1 was considered unlikely, as the stimulation target was the right paramedian motor cortex and the complaint developed multiple weeks following treatment. In contrast, stimulation target in subject 3 was the left paramedian motor cortex. Pain developed < 1 week after treatment, but not in immediate temporal relationship with cTBS delivery. Therefore, relation to cTBS was considered uncertain.

Subject 4 reported a few episodes of dizziness, which were also described during baseline and thus considered not related to cTBS. This subject also reported fatigue and a single episode of tremor and dizziness, of which the relation to stimulation for both was uncertain.

### Transcranial magnetic stimulation-related seizures

#### Clinical inspection

During cTBS delivery, no clinical seizures occurred. In two subjects (subject 1 and subject 3) habitual reflex myoclonic seizures occurred shortly following stimulation or in between two stimulation trains in subject 1. These were no different from habitual reflex myoclonic seizures that occurred at a high baseline frequency. Therefore the occurrence was considered unrelated to cTBS.

#### Online EEG monitoring

Evaluation of EEG, acquired during cTBS treatment sessions, did not identify the occurrence of electrographic seizure activity during or in between stimulation trains.

#### Seizure diary

Median number of seizures during the treatment week (6; IQR 14) did not differ significantly from baseline monitoring (3; IQR 7.5; *p* = 0.149; see Table 3). Single subject assessment showed an absolute number of seizures during treatment week that remains in the range of seizure frequency variation during baseline in all but one subject. This particular subject (subject 4) reported 12 seizures during the treatment week, whereas the number of seizures per week during baseline ranged from 0 to 5. Nine out of the 12 reported seizures consisted of short-lasting auditory phenomena of a few seconds, similar to the habitual seizure semiology, but of shorter duration than normal.

**TABLE 3:** Seizure frequency assessment.

														Median number of			Ratio of seizure		Response
Subject	Clinical semiology	Number of seizures												seizures per week			free weeks		rate
		B-4	B-3	B-2	B-1	Treatment	FU1	FU2	FU3	FU4	FU5	FU6	FU7	Baseline	Treatment	Follow-up	Baseline	Follow-up	Response rate total
1	Focal motor seizure of the left leg or left hemisoma	0	2	0	1	1	1	0	1	1	1	1	0	0,5	1	1	0,50	0,29	+100%
2	Focal motor seizure of the right trunk and arm	4	1	0	1	0	0	0	1	0	0	1	0	1	0	0			
	Focal motor seizure of the right hemisoma	3	3	2	1	0	0	1	2	0	2	3	2	2,5	0	2			
	Focal motor seizure with impaired consciousness and postictal paresis	0	0	0	0	0	0	0	1	0	0	0	0	0	0	0			
	All seizures	7	4	2	2	0	0	1	4	0	2	4	2	3	0	2	0,00	0,29	−33%
3	Reflex myoclonia of the right leg	9	37	11	4	26	13	6	1	1	0	1	13	10	26	1			
	Focal motor seizures of the right leg, onset during sleep	0	0	0	0	0	0	1	0	0	0	9	0	0	0	0			
	All seizures	9	37	11	4	26	13	7	1	1	0	10	13	10	26	7	0,00	0,14	−30%
4	Focal auditory seizures	3	2	0	5	12	1	3	1	4	3	1	1	2,5	12	1	0,25	0,00	−60%
5	Tonic seizure of right arm, often clustered	7	4	5	7	3	5	5	5	3	3	6	1	6	3	5	0,00	0,00	−17%
6	Nocturnal focal motor seizure of right arm and leg	12	10	6	24	15	5	7	2	6	8	18	12	11	15	7	0,00	0,00	−32%
7	Focal sensorimotor seizures of right hemicorpus	4	0	1	7	6	5	6	4	1	0	7	4	2,5	6	4	0,25	0,14	+60%
**All**	***median***													**3**	**6**	**4**	**0,00**	**0,14**	**−30%**
	***IQR***													**7,5**	**14**	**6**	**0,25**	**0,29**	**93**
	2-sided sign.*														***0,128***	***0,149***		***0,893***	

*Wilcoxon matched-pairs signed-ranks test (compared to baseline).

Blue represents baseline, orange represents treatment, green represents follow-up period, and grey is the percentage of seizure in, or decrease between baseline and follow-up period.

The bolded numbers represent the significant values.

#### Cognition

Results are reported in Table 4. Median MoCA score at baseline was 26 (IQR 6), without significant change following the treatment week (27; IQR 8) or at the end of follow-up (26; IQR 4; *p* = 1.000). On the single subject level, MOCA score increased in 3/7, decreased in 3/7, and remained stable in 1/7 subjects. There was no indication that any of the cognitive subdomains was selectively affected.

**TABLE 4:** Cognitive assessment.

	T0		T4		FU2		FU7		Statistics*
	Median	IQR	Median	IQR	Median	IQR	Median	IQR	*2-sided sign.*
**MoCA**									
(/30)	26.00	6.00	27.00	8.00			26.00	4.00	*1.000*
Visuospatial/executive (/5)	4	1	4	1			5	2	*0.943*
Naming (/3)	3	0	3	0			3	0	*0.607*
Attention (/6)	6	1	6	1			6	2	*0.257*
Language (/3)	2	2	2	3			2	1	*0.368*
Abstraction (/2)	2	1	2	0			2	0	*0.097*
Delayed recall (/5)	5	2	5	4			4	1	*0.638*
Orientation (/6)	6	0	6	1			6	0	*0.368*
**CVST**									
Time (s)	14.91	12.61	11.59	12.34	12.2	9.07	11.79	7.39	*0.059*
Error (n°)	2	3	2	2	0	3	2	3	*0.554*

*Friedman test.

MoCA, montreal cognitive assessment; CVST, computerized visual searching task; and IQR, interquartile range.

Median response time of the CVST at T0 was 14.91 s (IQR 12.61), at T4 11.59 (IQR 12.34), at FU2 12.20 (IQR 9.07), and at FU7 11.79 (IQR 7.39). Statistical analysis revealed a trend toward significance (*p* = 0.059). Individual response times improve over time in 6/7 subjects. The number of errors did not change throughout the study (*p* = 0.554).

#### Psycho-emotional well-being

Results are summarized in Table 5. Quality of life assessment revealed no significant change in overall calculated or subjective QOLIE-31 score at group level (*p* = 0.866 and *p* = 0.595, respectively). When individual scores were evaluated, subjectively scored QoL was improved in 4/7, remained stable in 1/7, and decreased throughout the study in 2/7 subjects (with 40 points for subject 3 and 10 points subject 7). The calculated QoL score only showed deterioration in one subject (subject 7), with a reduction of 16 points. In subject 3, who subjectively perceived a QoL deterioration of 40 points, the calculated QOLIE-31 score differed only by two points before and after cTBS.

**TABLE 5:** Psycho-emotional assessment.

	T0		T4		FU2		FU7		statistics*
	Median	IQR	Median	IQR	Median	IQR	Median	IQR	*2-sided sign.*
**QOLIE-31 calculated score (/100)**	49.89	13.20					55.12	22.03	*0.866*
**QOLIE-31 subjective score (/100)**	60.00	30.00					60.00	50.00	*0.595*
Seizure worry (/100)	47.32	27.68					50.00	33.02	*0.612*
Quality of life (/100)	72.50	22.50					67.50	27.50	*0.598*
Emotional well-being (/100)	64.00	20.00					76.00	32.00	*0.395*
Energy/fatigue (/100)	55.00	20.00					45.00	25.00	*0.891*
Cognition (/100)	56.12	40.29					39.72	43.61	*0.463*
Medication effects (/100)	47.20	29.70					55.57	36.13	*0.553*
Social function (/100)	22.00	16.00					31.00	32.00	***0.034***
**BDI (/63)**	8.00	12.00					10.00	12.00	*1.000*
**STAI**									
State (/80)	28.00	13.00					30.00	30.00	*0.173*
Trait (/80)	38.00	12.00					41.00	9.00	***0.041***
**PANAS**									
Positive affect (/50)	32.00	8.00					30.00	6.00	*0.674*
Negative affect (/50)	21.00	12.00					17.00	16.00	*0.752*
**VAS well-being (/100)**	70.00	25.00					60.00	30.00	*0.518*

*Wilcoxon matched-pairs signed-ranks test.

QOLIE, quality of life in epilepsy; BDI, beck depression inventory; STAI, state-trait anxiety inventory; PANAS, positive affect negative affect scale; VAS, visual analog scale; and IQR interquartile range.

Bold values are the significant values.

Assessment of the different QoL subscores reveals a significant improvement in social functioning (*p* = 0.034), with a median score of 22/100 (IQR 16) at baseline increasing to 31/100 (IQR 32) at final follow-up. On the individual level, social functioning was considered improved in 5/7 and stable in 2/7 subjects.

Depression assessment (BDI-II) revealed no significant change throughout the study (*p* = 1.000). Median group score at both time points was consistent with minimal depression (score 0–13; Hubley, 2014). On the individual level, all but one subject reported scores consistent with *minimal depression* (score < 13). One participant (subject 4) reported a score of 44/63 at baseline, compatible with *severe depression*, which decreased to 38/63 at FU7, which was still within the range of *severe depression*. This subject was concomitantly treated with antidepressants and followed by a psychiatrist prior to inclusion in the study. BDI-II score remained unchanged in 1/7, decreased in 2/7, and increased in 4/7. In subjects showing an increase, only one subject (subject 7) increased from *minimal* (score 9/63) to *mild depression* (score 14/63).

State of anxiety, scored using the S-STAI, was unaffected by cTBS (*p* = 0.173). One participant (subject 4) reported scores above the cut-off point of clinically significant symptoms at baseline, which remained stable throughout the study (58/80 and 59/80, respectively; Spielberger, 1983). Group score for anxiety trait (T-STAI) increased by three points, from 38/80 to 41/80, which appeared to be a significant change (*p* = 0.041). On the individual level, all but one subject showed an increase in T-STAI score, ranging from 2 to 7 points.

On a group level, both scores for PANAS were unaffected by cTBS (*p* = 0.674 and *p* = 0.752, respectively). Looking at individual scores, positive affect increased in 2/7 subjects, decreased in 3/7, and remained stable in 2/7. Negative affect decreased in 1/7 subjects, increased in 2/7, and remained stable in 4/7.

Median VAS score for well-being was unaffected by cTBS (*p* = 0.518). On the individual level, VAS score increased in 3/7, decreased in 3/7 and remained stable in 1/7.

### Feasibility/tolerability

**FIGURE 2:**
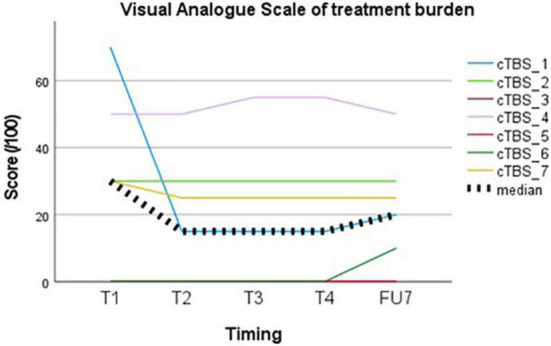
Visual representation of treatment burden perceived by the participants.

Treatment burden, evaluated using a VAS, showed a median group score of 20/100 (IQR 30) at the end of follow-up (see Figure 2). The highest score was given following the first session (30, IQR 50) and decreased thereafter (15, IQR 30).

None of the subjects withdrew from the study prematurely. Four out of 7 subjects (subject 1, subject 2, subject 3, and subject 5) were willing to repeat the treatment, especially if this would be associated with ongoing seizure suppression in between sessions. Two subjects were unwilling due to lack of perceived effect (subject 4 and subject 7) and one due to traveling distance (subject 6).

### Anti-epileptic effect

**FIGURE 3:**
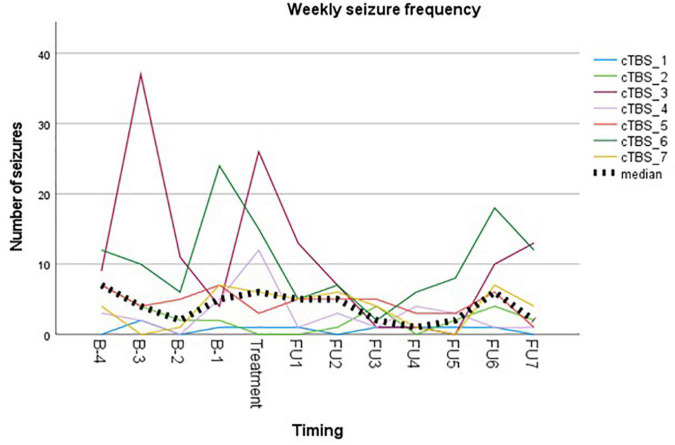
Visual representation of weekly seizure frequency. Colored lines represent weekly seizure frequency per subject. Bold dotted line represents the median group value.

Reported seizure frequencies and derived outcome parameters are summarized in Table 3 and Figure 3.

Myoclonic seizures in subject 1 were not included in the analysis due to unreliable reporting during follow-up after week 6. This is unfortunate, since the subject reported a complete abolishment of this seizure type for 6 weeks following cTBS, compared to 11 myoclonic seizures during the baseline period. Subject 3 reported a temporary leave from work from the second baseline week up until the third follow-up week. As stress and fatigue are known triggers in epilepsy, this period of relative rest may have biased evaluation of treatment effect. Subject 4 did not unambiguously report different seizure types/severities throughout the study, resulting in reporting them all under the general nominator of focal auditory seizures. The occurrence of prolonged episodes of speech difficulty during baseline was not logged reliably and therefore not included in the analysis. Subject 6 reported two clusters of seizure exacerbation during follow-up, occurring in the context of medication non-compliance. These seizures were omitted from the analysis. The subject denied non-compliance at any other time throughout the study.

Group analysis showed no difference in median weekly seizure frequency following treatment (*p* = 0.149), nor in ratio of seizure-free weeks (*p* = 0.893). Five out of seven subjects showed a reduction in median weekly seizure frequency, with a response rate ranging from −17% to −60%. Median response rate at group level was a 30% reduction in seizure frequency. Two subjects showed an increase in seizure frequency following cTBS (subject 1 and subject 7). Subject 1 showed a 100% increase in the occurrence of focal tonic seizures, increasing from 0.5 to 1 seizure per week (median value). However, these seizures were reported to be less severe in the initial 2 weeks following stimulation, in addition to a complete abolishment of his second seizure type (reflex myoclonic seizures) for 6 weeks following cTBS as previously described. Seizure frequency increased with 60% in subject 7 without impact on seizure severity.

## Discussion

### Safety of continuous theta burst stimulation in drug-resistant epilepsy

Our study is the first to apply an intensified TBS protocol in epilepsy patients. All patients completed the full study protocol. No serious adverse events were reported in our population. No clinical or electrographic seizures were acutely induced by cTBS.

**FIGURE 4:**
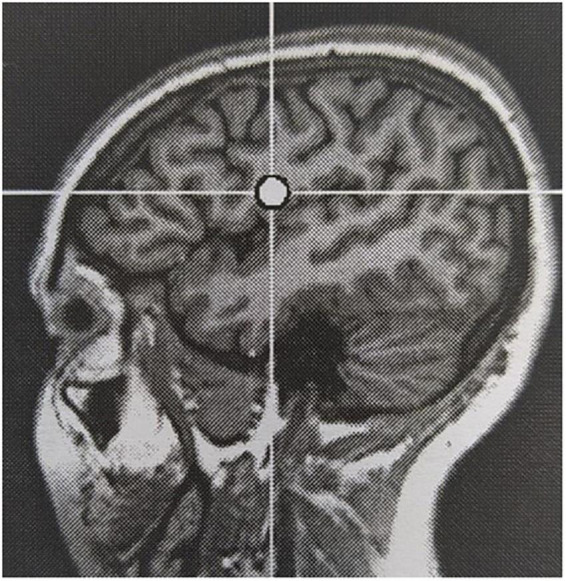
Stimulation target in subject 7.

Reported adverse events are generally in line with those reported using conventional rTMS in an epileptic population, but the incidence in our cohort was significantly higher (100% versus 18.3%; Pereira et al., 2016). All subjects reported local sensations underneath the stimulation coil during stimulation. Six out of seven subjects reported headache at some point during the evaluation, of which the causal relation to TBS being more likely in cases where it occurred in close temporal relationship to cTBS delivery. Headache was most often mild and short-lived, but required first line pain medication in 50% of subjects. Auditory adverse events, mainly sensory hearing damage, are well-known in TMS literature and addressed using adequate hearing protection during rTMS sessions (Rossi et al., 2021). One subject in our cohort reported hyperacusis, which extended a few days beyond the treatment week. She did not report pre-existent acoustic complaints. Worsening of a pre-existent hyperacusis is described in subjects treated for tinnitus following stimulation of the auditory cortex (Lefaucheur et al., 2012). The targeted EF in this subject was located in the left inferior frontal lobe, rather close to the auditory cortex across the lateral sulcus (see Figure 4). Therefore, the adverse event was considered likely related to TBS, possibly due to unintended co-stimulation of the auditory cortex. In analogy, the stimulation-induced sensory phenomenon in the right hemisoma in subject 3 during 2 out of 4 stimulation sessions was also considered related to TBS, as a result of co-stimulation of the somatosensory cortex adjacent to the stimulation target. Jaw contractions during stimulation occurred in two subjects. Stimulation target in these subjects were the auditory cortex and inferior frontal gyrus. Jaw contractions are the result of direct activation of nerve and muscle fibers by the magnetic field. Although the mechanism is unclear, dizziness in short bouts during stimulation in one subject was considered related to stimulation. In contrast, episodes of dizziness during follow-up in another subject were not. Two subjects reported a sensation in the hemisoma, contralateral to the stimulation site, during and immediately following stimulation in one and the morning after a stimulation session in the other. Both reported to recognize this sensation as a prodromal phenomenon, without the occurrence of a seizure. Online EEG monitoring did not identify the occurrence of any ictal discharges.

There is ample experience with TBS in non-epileptic subjects. Compared to conventional rTMS, there is no increased risk of seizure induction provided general safety recommendations are in place (Rossi et al., 2021). Experience with TBS in epilepsy patients is scarce and caution seems warranted in a seizure prone population. Koc et al. (2017) safely applied single cTBS trains over the motor and cerebellar cortex of 15 subjects with idiopathic generalized epilepsy. Udupa et al. (2020) performed TBS in seven epilepsy patients admitted to an epilepsy monitoring unit in an attempt to induce habitual seizures to speed up the diagnostic and localizing process. They applied both an excitatory iTBS and an inhibitory cTBS train over the EF, but were unable to induce any seizure activity, thereby concluding that TBS can be safely applied in this population. In our population, no clinical or electrographic seizures were evoked during cTBS sessions. A few myoclonic jerks occurred in close temporal relation to stimulation, but these were not different from habitual semiology nor was the frequency. On the group level, seizure frequency during the stimulation week did not differ significantly from absolute weekly seizure frequency values during baseline. However, in one participant the number of reported seizures during the stimulation week did exceed baseline weekly seizure frequency range, warranting caution. She experienced 12 auditory seizures compared to 0 to 5 events during baseline. However, nine seizures were of much shorter duration compared to habitual semiology and the situation was therefore not perceived as a deterioration of seizure control. Moreover, during follow-up, median weekly seizure frequency was reduced by 60% compared to baseline.

Safety assessment also comprised cognitive and psychological evaluation. In view of the bulk of the literature using mostly excitatory rTMS protocols to treat diverse neuropsychiatric conditions, our main intent was to monitor for a negative impact of an inhibitory cTBS paradigm over the EF on cognition and psycho-emotional well-being. In contrast, Sun et al. (2012) reported significant improvement on multiple subdomains of the Symptom Checklist-90 following low-frequency rTMS in epilepsy patients. We did not identify a negative impact of cTBS on MOCA, CVST or any of the psycho-emotional questionnaires. CVST response time showed a trend toward improvement over the course of the study (*p* = 0.059), but a learning effect cannot be excluded here. QOLIE-31 subscore on social functioning improved significantly on the group level (*p* = 0.034), from 22/100 (IQR 16) to 31/100 (IQR 32). T-STAI score, representing the anxiety trait of our subjects, appeared to increase significantly following stimulation (*p* = 0.041), but only with three points, of which the clinical relevance is unclear. Moreover, T-STAI score characterizes anxiety “proneness” as a longstanding trait or characteristic in a subject, which is expected to remain unaffected by intervention in contrast to the anxiety state, reflected by the S-STAI score which remained stable in our cohort.

### Feasibility of continuous theta burst stimulation as an anti-epileptic treatment

Treatment of DRE with cTBS seems feasible with regards to treatment burden. Tolerability of the stimulation itself was considered acceptable on the group level with a median VAS score of 20/100 (IQR 30) at the end of follow-up. Tolerance increases following the first session, which may reflect habituation to the TMS experience. There is a wide range of perceived AEs when individual scores are evaluated. This may be patient-related or related to different stimulation targets, with highest rates of annoyance, pain and muscle twitches reported in stimulation of frontal or inferior regions (Meteyard and Holmes, 2018). Treatment burden in our cohort was the highest in subject 4 (VAS 50–55/100), in whom the EF was located in the temporal region.

None of the subjects dropped-out and more than half of participants were willing to repeat treatment, especially if this would allow long-term seizure suppression. Lack of perceived effect was the reason not to repeat treatment in two subjects while traveling distance justified waiving repeated treatment in another one. Thus, feasibility of cTBS as a treatment for DRE would not only be dependent on a seizure reducing effect, but also on the duration of this effect and practicalities like traveling distance. Not unimportantly, such indirect stimulation-related factors (e.g., associated traveling costs and absenteeism) might negatively impact burden of cTBS in a chronic treatment setting.

### Anti-epileptic effect of continuous theta burst stimulation in focal neocortical drug-resistant epilepsy

The design and sample size of this open-label pilot study is unfit to reliably identify a therapeutic effect of cTBS in DRE. Nevertheless, results encourage the exploration of cTBS as an anti-epileptic treatment and potential optimization compared to conventional rTMS.

Out of seven subjects, five show a reduction in median weekly seizure frequency. Subject 1 is annotated as a non-responder in Table 3, based on the frequency of his reported tonic seizures. However, reflex myoclonic seizures, frequently leading to unexpected falls, were completely abolished for a duration of 6 weeks following treatment compared to a median weekly baseline frequency of 3.5. Unfortunately, this effect could not be included in the analysis due to unreliable reporting of the frequency of these seizures after their recurrence. Subject 7 showed an increase in median weekly seizure frequency by 60% during follow-up. Seizures were unaffected in severity. The range of absolute number of seizures per week during follow-up did not exceed that of baseline, but caution is warranted in future studies, preferably designed with longer and balanced baseline and follow-up periods.

Visual representation of the weekly seizure frequency (Figure 3) shows a maximal reduction in seizure frequency occurring between week 3 and week 5 following stimulation. This is in contrast to what is expected based on the meta-analysis on anti-epileptic effects of low-frequency rTMS (Mishra et al., 2020), but in line with the delayed antidepressant effect following accelerated iTBS reported by Duprat et al. (2016).

Practical advantage of TBS over conventional rTMS is evident in regard to the duration of the treatment session itself. Adequate duration of the anti-epileptic effect seems primordial in the acceptance of cTBS as a treatment. Repetition every few weeks is not feasible, for patients nor for clinical practice. Further optimization of stimulation parameters should strive for increased efficacy and duration of the effects. Such an attempt recently led to Stanford Neuromodulation Therapy, previously called Stanford Accelerated Intelligent Neuromodulation Therapy, in which 10 iTBS trains are applied daily for five consecutive days with a 50-min intertrain-interval and at 90% rMT (Cole et al., 2020, 2022). This protocol has shown potent anti-depressant effects, but superiority in efficacy or durability remains to be proven. Moreover, the application of a more heavily loaded stimulation protocol might not be the optimal solution. Accumulating evidence points toward concepts like homeostatic metaplasticity, cortical state-dependency and dynamic brain oscillations to impact the after-effects of neurostimuation (Zrenner et al., 2018; Thomson and Sack, 2020; Leodori et al., 2021), requiring a patient-tailored approach rather than a “one-size fits all” stimulation protocol. The development of state-dependent real-time EEG-triggered TMS may allow to reduce variability in the after-effects of cTBS and as such optimize its neuromodulatory outcome.

Finally, superior effect of rTMS is achieved in neocortical epileptogenic lesions, which is only a limited subset of the patients with DRE. The effect of rTMS on deep foci is probably insufficient, based on the physical limitations of the magnetic field strength that falls rapidly with increasing distance from the stimulation coil. Specifically designed stimulation coils allows for an increased penetration depth. This is, however, at the expense of stimulation focality (Lu and Ueno, 2017) and experience in the context of epilepsy is sparse (Gersner et al., 2016). To affect deep epileptogenic lesions, targeting of cortical areas functionally connected to deeper foci is under investigation (Shafi et al., 2015). Superficial areas may act as a “cortical window,” in analogy to targeting the DLPFC that connects to deeper subgenual cingulate cortex in rTMS for depression. Proof-of-concept with regards to the anti-epileptic effects of this “window” approach remains to be provided.

## Conclusion

We conclude to encouraging results of the first ever trial of cTBS as a treatment for neocortical DRE. A 4-day accelerated cTBS protocol was not associated with major AEs, nor were seizures induced. The anti-epileptic effect of cTBS could not statistically be confirmed, but further research seems warranted in a dedicated randomized controlled trial. The aim is to further optimize treatment efficacy, durability and practicality, prerequisites to position cTBS within the therapeutic arsenal of the clinical epileptologist.

## Data availability statement

The raw data supporting the conclusions of this article will be made available by the authors, without undue reservation.

## Ethics statement

The studies involving human participants were reviewed and approved by Ethics Committee of Ghent University Hospital. The patients/participants provided their written informed consent to participate in this study. Written informed consent was obtained from the individual(s) for the publication of any potentially identifiable images or data included in this article.

## Author contributions

SC, PB, EC, MM, RR, and KV contributed to conception and design of the study. SC performed participant screening and inclusion, supported by AV and AM. SC and DK conducted the data acquisition. KV performed EEG analysis. SC organized the database and performed (statistical) analysis, and wrote the first draft of the manuscript. All authors contributed to manuscript revision, read, and approved the submitted version.
